# Status does not predict stress: Women in an egalitarian hunter–gatherer society

**DOI:** 10.1017/ehs.2020.44

**Published:** 2020-08-24

**Authors:** Piotr Fedurek, Laurent Lacroix, Julia Lehmann, Athena Aktipis, Lee Cronk, Cathryn Townsend, E. Jerryson Makambi, Ibrahim Mabulla, Volker Behrends, J. Colette Berbesque

**Affiliations:** 1 Centre for Research in Evolutionary, Social and Inter-Disciplinary Anthropology, Roehampton University, London, UK; 2 Health Sciences Research Centre, Roehampton University, London, UK; 3 Department of Psychology, Arizona State University, Tempe, AZ, USA; 4 Department of Anthropology, Baylor University, Waco, TX, USA; 5 Mount Meru Tour Guide and International Language School, Arusha, Tanzania; 6 National Museums of Tanzania, Dar es Salaam, Tanzania

**Keywords:** cortisol, egalitarianism, hierarchy, hunter–gatherers, prestige, social status

## Abstract

It is widely believed that there is strong association between physiological stress and an individual's social status in their social hierarchy. This has been claimed for all humans cross-culturally, as well as in non-human animals living in social groups. However, the relationship between stress and social status has not been explored in any egalitarian hunter–gatherer society; it is also under investigated in exclusively female social groups. Most of human evolutionary history was spent in small, mobile foraging bands of hunter–gatherers with little economic differentiation – egalitarian societies. We analysed women's hair cortisol concentration along with two domains of women's social status (foraging reputation and popularity) in an egalitarian hunter–gatherer society, the Hadza. We hypothesized that higher social status would be associated with lower physiological indicators of stress in these women. Surprisingly, we did not find any association between either foraging reputation or popularity and hair cortisol concentration. The results of our study suggest that social status is not a consistent or powerful predictor of physiological stress levels in women in an egalitarian social structure. This challenges the notion that social status has the same basic physiological implications across all demographics and in all human societies.

## Graphical abstract

**Figure:**
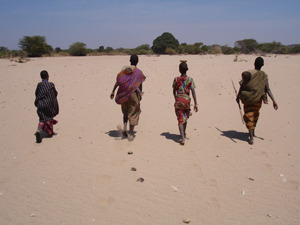

**Media Summary:** By analysing women's stress in egalitarian hunter–gatherers, we find that social status does not have the same impact across human societies.

## Introduction

The biosocial model of status, in which every member of a social group signals social status through behavioural signs (e.g. demeanour, speech, physical conflict) (Mazur, 1985) is a model that has relied far more on research on males than females. Despite the fact that the biosocial model of status is considered a universal in social animals, there are some fundamental gaps in the literature on social status and stress. First, most of the literature on social status and both perceived stress levels and physiological stress levels in humans comes from large-scale, industrialized populations. These typically use socioeconomic status (operationalized as income and education) as a proxy for social status (Marmot, 2004; Sapolsky, 2004; Wilkinson, 2001). However, the vast majority of human evolutionary history was spent in small bands of hunter–gatherers with little economic differentiation between individuals (Bowles et al., 2010; Mattison et al., 2016), in contrast to the large-scale social and economic interactions that characterize industrialized populations. Furthermore, there is very little research on social status and stress in small-scale populations. There are a few very valuable papers on social status and stress in hunter–horticulturalist men (Konečná & Urlacher, 2017; Trumble et al., 2014; von Rueden et al., 2014), but there are no data on hunter–gatherer populations. Hunter–gatherer populations are distinct from other small-scale societies in that they have well-documented, explicit levelling mechanisms to prevent hierarchy, rather than simply an absence of central authority (Boehm et al., 1993; Cashdan, 1980).

The second gap in the literature on social status and stress is reflected in its almost exclusive focus on men or on mixed-sex groups (Hamilton et al., 2015; Konečná & Urlacher, 2017; Trumble et al., 2014; von Rueden et al., 2014). To date, little is known about the effects of social status on individual stress levels in women's groups. The link between social status and cortisol profiles established in males or mixed-sex groups has merely been assumed to be the same for female social groups, with very little actual investigation to date (Casto & Prasad, 2017).

This lack of data on women's social status and stress is a critical oversight given that, in non-human primates, stress and social status can have important impacts on reproductive fitness. In addition, social status is often manifested in female-only hierarchies. In our closest relatives, chimpanzees, females with lower social status have higher cortisol levels (Emery Thompson et al., 2010) and socially dominant females have greater reproductive success than lower-status individuals (Pusey et al., 1997; Wittig & Boesch, 2003).

In the literature, social status has been considered to be multidimensional, including traits such as physical formidability, material resources and socioeconomic status (Lukaszewski et al., 2016). Human status can also be based on prestige, or freely conferred deference from others (Henrich & Gil-White, 2001; Cheng & Tracey, 2013). Studies in WEIRD (Western, educated, industrialized, rich and democratic) societies (Henrich et al., 2010) have shown that individuals with higher social status based on prestige, rather than money, live longer than economically equal peers without prestige (Liu et al., 2017; Redelmeier & Singh, 2001).

Likewise, ethnic minority status is associated with higher cortisol levels, independent of socioeconomic status, even in an environment with little disparity in access to resources (e.g. the Netherlands) (Rippe et al., 2016). Self-perceived status also appears to be important in shaping physiological responses to stress. When exposed to a laboratory stressor, individuals with lower self-perceived social status had more pronounced inflammatory responses compared with those that perceived themselves to be of higher status. This effect remained significant, even when controlling for actual differences in socioeconomic status (Derry et al., 2013).

Popularity can be also regarded as a measure of social status (Kindermann & Gest, 2009; Kornienko et al., 2013). Previous studies have shown that low levels of nominated friendship might be related to social anxiety resulting from a self-perceived social rejection or withdrawal (La Greca & Lopez, 1998) and some studies have shown that the latter is related to elevated levels of physiological stress (Granger et al., 1994, 1996). It has also been shown that individuals who are perceived as popular have greater access to social support compared with less popular individuals (Kornienko et al., 2013).

On the other hand, in a study of women's networks, being very popular (i.e. being a recipient of a relatively high number of friendship nominations) can also have adverse effects on psychological wellbeing of an individual potentially driven by resentment, jealousy and, most of all, a chronic fear of status loss which may involve constant monitoring of social relationships (Kornienko et al., 2013). Indeed, being on top of the social hierarchy of social animals is often associated with unstable social relationships (Sapolsky, 2005) and, therefore, higher stress levels (Gesquiere et al., 2011). Moreover, one human study found that high turnover in friendship relationships is related to higher stress levels (Kornienko et al., 2016).

Ironically, it is not easy to choose a dimension of social status in the Hadza, because they are extremely egalitarian. There are no differences in personal property or wealth or leadership within women. Even body condition among each sex is relatively homogeneous (Sherry & Marlowe, 2007). Status-seeking by either gender is actively discouraged, and several levelling mechanisms are used to curtail self-aggrandizing and status-seeking behaviour. These levelling mechanisms in hunter–gatherer societies are fully described by Woodburn (1982, 2005) and others (Boehm et al., 1993; Cashdan, 1980).

We chose foraging reputation as a second possible proxy for social status for several reasons. First, the Hadza have reported foraging ability as an important quality in a mate for both genders (Marlowe, 2004). Second, hunting reputation in males is described as both being valid and reliable (Stibbard-Hawkes et al., 2018) and also as a prestige-conferring activity that translates into higher reproductive success (Apicella, 2014). However, men with better hunting reputations do not have wives with better nutritional status (Stibbard-Hawkes et al., 2020), which suggests widespread food sharing. In addition, women's production of tubers as fallback foods may actually reduce inequality in access to calories, even if meat is not equally shared (Marlowe & Berbesque, 2009). While digging reputation is not the same as hunting reputation, it does make sense to measure women's most obvious contribution to subsistence. We expect that women who produce more might have more opportunity to garner prestige through distribution or through the reputation of being a hard worker.

There have been few studies on social status in women in non-industrialized societies, but a study of 33 non-industrialized societies found measurable differences in the social status of men regardless of the ‘political egalitarianism’ of their societies, with status measured by indicators such as wealth, leadership, hunting and culturally specific status indicators (von Rueden & Jaeggi, 2016). However, many of the indicators used in this study (such as wealth and leadership) are absent in egalitarian hunter–gatherer societies such as the Hadza, and none of the indicators are relevant for Hadza women. We argue that this is because the Hadza are far more egalitarian than the non-industrialized societies that have been documented to a large extent. It is noteworthy that there has been one study in the very egalitarian BaYaka hunter–gatherers that finds that, in both sexes, having more relational wealth is associated with higher body mass index and relational wealth is associated with higher age-specific fertility in women (Chaudhary et al., 2016). Relational wealth varied far more in men than in women among the BaYaka. This pattern was also documented in Agta hunter–gatherer women, where women with more second- and third-degree ties (indirect centrality) in a social network had more surviving children (Page et al., 2017). However, these findings may be due to differences in social support in egalitarian populations rather than social status per se. While social support may confer status or influence social position in some cultures, it is not a proxy for social status. An individual with greater social support may not cause others to evaluate them more favourably, and some types of ‘social support’ may even negatively impact an individual (e.g. Boutin-Foster, 2005).

Before claiming that social status is a critical factor for determining health in all human populations, we must document that status differentials affect women as well as men and are present cross-culturally – including in the most egalitarian contexts. To this end, we assess the relationship between long-term cortisol levels and reputation for two potential domains of women's social status (foraging reputation and popularity) in the Hadza, hunter–gatherer women.

## Methods

### The Hadza People

The Hadza are traditionally hunter–gatherers, and now number approximately 1000. They live in a savanna-woodland habitat that encompasses about 4000 km^2^ around Lake Eyasi in northern Tanzania. Approximately 250 Hadza are still living traditionally from mostly foraged foods. They live in mobile camps, which average 30 adult individuals. Camp membership often changes as people move in and out of them. These camps move about every 6 weeks on average (Marlowe, 2010; Jones, 2016). Hadza women hunt and gather other resources, foraging on average 4 hours per day. Although the Hadza often live in nuclear family units, women and men have separate spaces for socializing and working while in camp. Women with small children and babies often sit together in a shady spot doing beadwork, or work together processing foods. Hadza hunter–gatherer women forage together in groups and have separate physical areas from men in camp. Men also have their own space, where they work on arrows or bows (Marlowe, 2010; Jones, 2016). Hadza women spend a great deal of time exclusively with other women. Separate social spheres for each sex are common in many societies, including small-scale societies, but also in some industrialized contexts such as Europe and the US (Kerber, 1988; McBain, 1990). In some human societies, female social hierarchies may be entirely separate from men's hierarchies, and this is also common in non-human primates (Caspari, 1978).

The Hadza are politically egalitarian, with no official big men, chiefs or leaders (Woodburn, 1982; Woodburn, 2005). The Hadza also have very little disparity in material wealth, generally owning only personal possessions such as clothing (often a single outfit), bows and arrows (owned by men only) or digging stick (owned by women only), and knives (typically owned by men but also sometimes by women). In some cases, there is communal property used by a multigenerational family, such as a large cooking pot. Some individuals may possess items above and beyond these, but often these are still shared. One example of this is a machete, which not all Hadza men own and almost no Hadza women own. A machete owned by a particular man may be used (borrowed) by other men if needed, and even by women in camp in order to sharpen their digging sticks. Most personal property is freely loaned or even borrowed without explicit permission: any individual's insistence on explicit permission would point out a difference in property, already in itself considered rude. Theft is very rare, and most individuals have access to the same array of private property – there are no real status benefits to owning a machete, for instance.

There are some common forms of inequity in relatively ‘egalitarian’ societies, for example greater access to preferred foods (Berbesque et al., 2011; Berbesque and Marlowe, 2009; Speth, 1990), so there is reason to suspect that there are substantial enough differences in social status that we might see impact on stress levels. Although there are no official leaders, there are gender differences in status, with Hadza men having slightly more access to external goods and money (e.g. wage labour) than Hadza women. Hadza women are free to choose their own husbands, and to divorce if they wish to, and they are often vocal and opinionated. However, they are sometimes subject to domestic violence and generally not encouraged to travel alone whether for visiting or foraging.

Among the women in our Hadza hunter–gatherer study sample, there is no evidence for inequities in health as measured by body condition (Sherry & Marlowe, 2007; Marlowe & Berbesque, 2009; Raichlen et al., 2017), nor are there any substantial individual differences in personal wealth (Marlowe, 2010). However, there may still be individual variation in other measures of social status (von Rueden et al., 2014), such as popularity (Apicella et al., 2012), or in competency in foraging (Berbesque et al., 2016; Fitzpatrick, 2018). The question here is whether these differences are substantial and consistent enough to result in predictable associations between status and cortisol, as seen in other species, and in men in hierarchical societies.

### Data collection

Data collection was conducted in 2016 and 2017 for roughly four months each. Data were collected in camps that were actively foraging for most of their calories, although visiting and some limited trade with neighbouring food-producing groups like the Datooga pastoralists did occur as it has in all camps since Hadza life has been documented. The Hadza generally do not know their birth year, so age was established through long-term records established by Nicholas Blurton Jones (Jones, 2016), and maintained by Frank W. Marlowe (Marlowe, 2010) and JCB. Data collection lasted several weeks in each camp, with hair sampling conducted on the final day of data collection. This meant that part of the hair sample grew during the time when camp members were co-resident with the other members they were nominating.

Hair samples and reputational data on popularity status and foraging reputation were collected from eight camps of different sizes (ranging from 6 to 38 adults, mean = 19). All women present in every camp participated (*n* = 83), but the number of women present differed in each camp, ranging from 3 to 21, mean = 10.75). Only adult individuals were included in the study. On average, we collected reputational data from 100% of adults per camp (83 women and 64 men) and hair samples from 77 of the same women. Of the 147 study participants, two men and three women were sampled twice as they happened to be in two different study camps when the study was conducted. The hair samples from the first camp only were used for the women sampled twice, but nominations provided in both camps were used (more detail below). Given that only approximately 250 Hadza are full-time foragers, and approximately half of those are men, this constitutes a robust sample of one of the last extant egalitarian hunter–gatherer populations.

### Reputation measures

To measure reputation for Hadza women's competency in a socially valued skill, we asked 147 adult camp members (both men and women resident in camp), in private, one at a time, who were the three best tuber diggers in camp. Tubers are the most challenging regularly targeted food item for women to acquire and an important fallback food for the Hadza (Marlowe & Berbesque, 2009). Both men and women only nominated women as the top three ‘best diggers’, which was expected, because men rarely dig for tubers. Reputation measures were collected on 83 women, with a mean age of 41.02 years (median = 37, range 18–89).

In the same interviews, we also asked all respondents (*n* = 147 adults, 83 women and 64 men) who were their three best friends in that camp. Although they were free to nominate any adult (including men), all 83 women but three named other women as their three best friends in camp, and only two men named a woman as a friend. This means that, although we asked 147 people who their friends were, only women (*n* = 83) could be included in the sample for friendship nominations.

Reputational data on friend status and digging status were generated by assigning 3 points to the top score (such as first best friend), 2 points to the second and 1 point to the third best nominee. Because we collected reputational data from five camps of different sizes with different numbers of potential nominators, we standardized these raw values by deducting a mean camp value from each individual score in a camp and then dividing it by the standard deviation of the camp – which resulted in a within-camp *z* score for each reputation metric for each woman (Kreyszig, 1979). In addition, we calculated the skewness of the nominations scores using the e1071 (Meyer et al., 2019) package for R (R development core team 2019), where positive and negative values indicate positive and negative skew, respectively.

### Cortisol analysis

We are mainly interested in chronic longer-term levels of stress rather than the acute stress response because we are interested in the mediation of chronic stress. Hair cortisol has the advantage of allowing back-tracking average levels of cortisol over a longer time-frame than other methods (such as salivary cortisol); depending on hair length and growth rate, cortisol can reflect stress levels experienced over the last few months (Wennig, 2000). Assuming an average hair growth rate of 1 cm/month (Wennig, 2000), the record of hair cortisol in our samples covered the last 1–2 months, which corresponds roughly to the time documented as the average duration of Hadza residence in a particular camp (Marlowe, 2010). Hair cortisol concentration (HCC) levels have been found to be associated with self-perceived stress and poorer self-perceived health and mental health in people living in industrialixed contexts (Faresjö et al., 2014; Stalder et al., 2017; Staufenbiel et al., 2013). In addition, there is a well-documented association between cortisol, insulin resistance, hypertension, immunosuppression and reproductive impairments – having higher cortisol levels is associated with serious health implications, including reduced life expectancy (Güder et al., 2007; Kumari et al., 2011; Marmot & Sapolsky, 2014).

Determining cortisol from hair also has the benefit of being a biological sampling procedure that causes minimal discomfort to the participants and samples do not require refrigeration after collection (which would be quite difficult in mobile, fluid bush camps). It is worth noting that many Hadza women keep their hair short, so that the time period of cortisol levels detected in hair is estimated to be approximately one month. The Hadza do not dye or bleach their hair. They occasionally use soap (generally hand soap) to wash their hair, so cortisol values were unlikely to be affected by frequent shampooing, as seen in other cultures (Hoffman et al., 2014).

### Hair preparation and analysis

Hair cortisol was extracted according to Sauvé et al. (2007). Briefly, hair samples from the 1 cm closest to the scalp end were cut into small pieces using sterile small surgical scissors (Slominski et al., 2015), weighed (to around 10–15 mg) and placed into 1.5 ml reaction tubes. Prior to extraction, hair samples were ground using the IKA Ultra Turrax Tube drive System (following Xiang et al., 2016). For extraction, 1.5 mL of methanol was added, and the vial was sealed and incubated overnight for 18 h at room temperature while gently shaking. After incubation, samples were centrifuged, the methanol extract was transferred to a disposable glass vial and evaporated to dryness under nitrogen. The samples were dissolved in 250 μL of phosphate buffered saline (pH 8.0). Samples were vortexed for 1 minute, and then again for 30 seconds before the assay.

Cortisol levels were measured using the Salimetrics® cortisol enzyme-linked immunoassay (ELISA) kit (Salimetrics Europe, Suffolk, UK) as per the manufacturer's instructions. In principle, the assay measures competitive binding to a capture antibody between hair-extracted cortisol and cortisol conjugated to horseradish peroxidase, which converts 3,3′,5,5′-tetramethylbenzidine to 3,3′,5,5′-tetramethylbenzidine diimine in a chromogenic reaction. After termination of the reaction by adding sulphuric acid, absorbance was measured at 450 nm and cortisol levels were calculated based on a standard curve. The intra- and inter-assay coefficients of variance were <9% and <10%, respectively.

### Statistical analysis

Cortisol concentrations were highly skewed, and thus we ln-transformed these measurements. After that, the visual inspections of normality and homogeneity of error variances did not indicate a violation of model assumptions.

We analysed the data using linear mixed-effects model (LMM) fit by the maximum likelihood estimation with cortisol levels as a dependent variable while foraging reputation and popularity status were used as predictors. Some of our study subjects that provided hair were pregnant (*n =* 4) or lactating (*n =* 14) and pregnancy and lactation have been found to affect physiological stress levels in women (Carr et al., 1981; Heinrichs et al., 2001). In order to control for these factors, we included in the LMM model an additional predictor – reproductive state – with two categories: (1) non-pregnant or lactating; and (2) pregnant or lactating. Regarding the three women that participated in the study twice in two different camps, for the LMM analysis we used only data from the camps they participated in first in order to avoid pseudo-replication (although their nominations were included in quantifying the popularity and digging status of other participants they nominated in both camps they participated in). We also included participant age in the model as a fixed independent variable and camp as a random variable. Because both digging status and popularity had quadratic relationships with age, we logged the age values (von Rueden et al., 2008).

In order to minimize the problem of collinearity, we first ran Kendal Tau correlations on all variable combinations and excluded highly correlated variables (Kendal tau > 0.8). We also calculated variance inflation factors (VIF) for all the variables, including only variables with VIF < 4. We calculated marginal (i.e. for fixed effects only) and conditional (i.e. for both fixed and random effects for camp) *R*^2^ for the LMM model using the ‘lmerTest’ package (Kuznetsova et al., 2017) for R. LMM was performed using the ‘lme4’package (Bates et al., 2014) for R (R Development Core Team, 2018). Because popularity and digging status correlated with each other (tau = 0.25, *p* = 0.001), we calculated two separate LMM models: one with all variables retained from the original model but without popularity and the other with all variables retained from the original model but excluding digging status (see Supplementary Material Tables S2 and 3). In addition, we used a Wilcoxon rank sum test in order to determine whether HCC values differed between the two field seasons (e.g. 2016 and 2017) during which the hair samples were collected.

## Results

**Figure 1.**
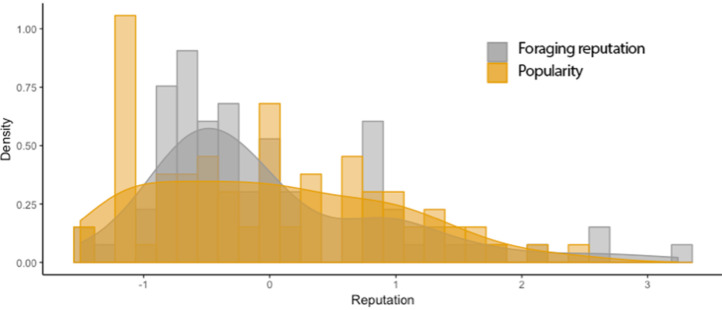
Histogram of reputations: foraging and popularity. The *x-*axis is the within-camp *z* score for each reputation metric for each woman.

Our cortisol sample consisted of 77 women, with mean hair cortisol concentration 78.25 pg/mg (median = 64.38, range 15.04–229.36). A Wilcoxon rank sum test found no differences in HCC between the two data collection field seasons (*W* = 764.5; *p* = 0.696). Both popularity and foraging reputation scores exhibited a positive skew, especially in bigger camps (Table A1, Supplementary Material). See Figure 1 for histograms showing the distribution of reputation measures (individual *z* scores), and Supplementary Figures S1a–h and S2a–h, showing some individuals received the majority of nominations for both measures in each camp.

Although some model variables were significantly correlated with each other (Table 1), Kendall tau and all VIF values were generally low (age VIF = 1.59, foraging reputation VIF = 1.80, popularity VIF = 1.25, reproductive state VIF = 1.08), thus all variables were included in the full model.

**Table 1.** Kendall correlation results between variables included in the model

	Foraging reputation	Popularity
Age	tau = 0.48, *p* < 0.001	tau = −0.008, *p* = 0.919
Foraging reputation		tau = 0.25, *p* = 0.0015

**Figure 2.**
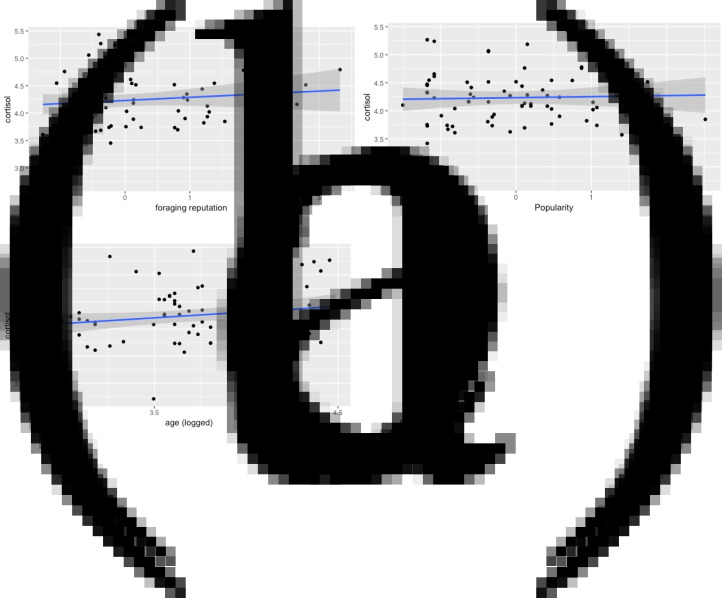
Foraging reputation, popularity and age associations with logged cortisol. Relationship between logged picograms of hair cortisol concentrations and within-camp *z* score for each reputation metric for each woman: (a) is foraging reputation, (b) is popularity and (c) is logged age. Shaded area represents 95% confidence intervals.

Neither age not foraging reputation nor popularity were associated significantly with hair cortisol concentrations (Table 2; Figure 2).

**Table 2.** Linear mixed-effects model results explaining cortisol concentration variance in the Hadza woman

	*Estimate* ± SE	*t*-Value	Pr(>|*t*|)	AIC	*R*^2^
Intercept	3.506 ± 0.52	6.72	<0.001	117.3	Marginal: 0.04
Age (logged)	0.195 ± 0.14	1.39	0.169		Conditional: 0.14
Popularity	0.017 ± 0.06	0.28	0.779		
Foraging reputation	−0.007 ± 0.07	−0.10	0.922		
Reproductive state	−0.073 ± 0.13	−0.56	0.575		

Neither popularity nor digging status were significantly associated with cortisol when these two variables were included separately for the LMM analyses (Tables S2 and S3, Supplementary Material).

## Discussion

Our results clearly indicated that, contrary to expectations, neither foraging reputation nor level of popularity had any effect on long-term stress levels in Hadza women. This lack of an association between foraging reputation or popularity and stress may result from (1) neither foraging ability nor popularity reflecting or conferring social status, (2) meaningful differences in social status not impacting on stress as measured via hair cortisol or (3) an absence of meaningful differences in women's social status.

The first possible explanation for our finding that Hadza women's popularity is not associated with their cortisol levels is that friendship nominations (or popularity) do not reflect social status. This finding is in contrast to recent findings in industrialized populations, where it has been shown that being frequently named as a close friend of others is associated with low physiological levels of stress in both adults (Holt-Lunstad et al., 2010; Kim et al., 2016; Ketay et al., 2017) and children (Peters et al., 2011; Kornienko et al., 2013). In addition, a nine-year study of children in a rural Caribbean village found no evidence that socioeconomic status per se affected the cortisol profiles or health outcomes of children; family relationships were more influential (Flinn & England, 1997).

Despite our findings, it is very likely that popularity is important among the Hadza, even if it has no predictable link with physiological stress. Indeed, computer models show that having friends that can be relied on in times of need can help buffer individuals from the risks of living in an uncertain ecological environment (Aktipis et al., 2016; Cronk et al., 2018). The importance of managing risk through building supportive relationships has been noted by other researchers studying small-scale societies. For example, Gurven's et al.'s (2000) work on the Ache notes that social relationships can function as a sort of social insurance for times when individuals may be in need. It is interesting that popularity does not predict cortisol levels across all human cultures, although it commonly does in WEIRD populations. It is possible that popularity confers social status as well as social support in hierarchical societies, but only provides support in a more egalitarian population.

Surprisingly, we also did not find any association between foraging reputation and hair cortisol levels. As tuber digging is a culturally valued skill (Marlowe & Berbesque, 2009), we expected that higher digging status would lead to lower cortisol levels. However, perhaps foraging reputation is not a good indicator of Hadza women's social status. Competition among women is associated with elevated cortisol in some WEIRD contexts (Casto & Edwards, 2016; Sherman et al., 2017). Among the Hadza, women are less likely to compete than men, even in gender-neutral tasks (Apicella & Dreber, 2015). This lack of competitiveness might explain the absence of an effect on cortisol.

On the other hand, even if women directly compete (our measures of status are indirect competition), winning a competition might not always be beneficial for an individual's social status. In WEIRD societies it may seem obvious that winning a competition or, as a form of indirect competition, being noticeably superior in some skill, would help an individual gain social status. That may not be the case in Hadza women's social spheres, where engaging in overt competition or even demonstrating superior skill may be frowned upon. There is a large amount of variation in attitudes towards competition in women cross-culturally. While women are often engaged in overt competition in some cultures, women are actively discouraged from overtly competing in other cultures (Andersen et al., 2012; Gneezy et al., 2009). Effectiveness in competitive contexts may even have the counter-intuitive effect of decreasing female status, particularly if women are denigrated by competitors (Sheppard & Aquino, 2017; Tichenor, 2011).

Finally, the common finding that women are less competitive than men in economics experiments may be at least in part due to the design biases. Experimental games designed to measure competitiveness may be framed in ways that do not offer the rewards most valued by women, thereby incentivising the efforts of women less than men. Gender gaps in competition were not supported in more recent games that allow participants to have some form of control over the decision of whether or not to share the prize with other players (Cassar & Rigdon, 2019) or to benefit the player‘s children rather than cash winner-takes-all prizes (Cassar et al., 2016). This suggests that, when women compete, they may do so more readily in different circumstances than men do, they may use different strategies than men to compete, and they may be driven by different goals than men (Liesen, 2013). Motivational differences between women and men might also affect how status is negotiated or achieved as well as how the social status of an individual is evaluated by each sex.

It is possible that both our indicators of social status in Hadza women have failed to actually capture meaningful differences in social status. Possible other factors contributing more to women's social status than the factors we describe in this study include attractiveness, helpfulness or intelligence. It is at least noteworthy that after many years (over a decade, in the case of JCB) of studying the Hadza, researchers are still uncertain what the best measure of social status in Hadza women would be.

The second possible explanation for our results is that, although there are meaningful differences in women's social status, they do not impact stress (as measured via hair cortisol). This would mean that differences in women's cortisol are not explained by social status as measured by foraging reputation or effectiveness in other competitive contexts. If this is the case, then variation in cortisol may be more related to other sources of stress (such as parasite load or nutritional stress).

The third possible explanation for why we did not find an effect of foraging reputation on cortisol is that it may be that too much status seeking (by any measure) is discouraged in an egalitarian society. In an extremely egalitarian society, individuals who overtly seek status may incur negative consequences. There are well-documented levelling mechanisms commonly used against individuals seeking high status in hunter–gatherer populations, which include: teasing, ostracism and insults (Boehm et al., 1993; Cashdan, 1980). Even if slight status differences exist among the Hadza women, these levelling mechanisms could dampen any physiological benefits of higher status.

Societies with the most extensive egalitarian practices and levelling mechanisms are sometimes referred to as non-competitive egalitarian societies, and these include, among others, the Hadza; the Batwa, BaYaka and Mbuti hunter–gatherers of Central Africa; and the Batek and Chewong hunter–gatherers of Malaysia (Woodburn, 1982; Townsend, 2018). In the most egalitarian societies, there appears to be a tendency to understate differences in ability between individuals and to value the diversity of skills that assorted individuals can bring to the group. For example, among the Batek, a recent study found that social levelling mechanisms, such as the cultural requirement to be humble, may suppress the potential relationship between status-seeking behaviour and reproductive outcomes. Four predictors of lifetime reproductive success – foraging return rate, sharing proclivity, cooperative foraging tendency and kin presence – could not explain variation in lifetime reproduction among males or females (Kraft et al., 2019).

Meanwhile, BaYaka hunter–gatherers recognize each other's skills and talents but ‘individual ability is downplayed and represented as a consequence of their conduct in relationships with other people and mystical agents (Lewis 2002: 251). According to Winterhalder's (1986) model, the risk-pooling benefits of sharing are maximal when there is variance in an individual's average resource return rate and lack of synchrony between the returns of individual foragers. Perhaps then it is unsurprising that extremely egalitarian cultures tolerate and even encourage variation in skills in order to enable social cohesion and the exploitation of a variety of resources. That said, many studies do find associations between measures of social status and reproductive success among men in even the most egalitarian societies (von Rueden & Jaeggi, 2016).

Our study provides the first data on social status and cortisol in hunter–gatherer women. Generally speaking, there is a large and unrealized opportunity to investigate social status and stress in female-exclusive social groups. Some researchers have suggested that female–female competition in female-exclusive status hierarchies in WEIRD societies have not garnered much attention possibly because workplaces that are exclusively female are rare (Mast, 2002). However, female-dominated (or even female-exclusive) work environments do exist and have done since the beginning of industrialization, if not before. Some industries have been dominated by women for quite a long time (e.g. ‘pink-collar jobs’, such as housekeepers, childcare providers, and retail clerks; Fortin & Huberman, 2002; Snyder & Green, 2008). Many other contexts involve hierarchies that are female exclusive, as in the so-called ‘Mommy Wars’, which by definition are a female-exclusive domain of competition (Crowley, 2015).

Even in workplaces where both sexes are present, ‘homosociality’, or the tendency to hire and socialize with only one's own sex, is fairly common (Elliott & Smith, 2004; Joyce & Walker, 2015; Schilt & Wiswall, 2008). This is true even in relatively egalitarian contexts. For example, Swedish male managing directors recruited other males almost exclusively, despite the fact that they considered themselves to be pro-gender equality and were aware of gender bias in hiring (Holgersson, 2013). In addition, a recent study found that, in the ethnographic literature, women's social capital (or women's reputation) has received far less attention than men's (Post & Macfarlan, 2020). The dynamics of female-exclusive status hierarchies or feminine forms of social capital can no longer be considered niche or peripheral to understanding how humans establish and maintain social status. Women's social status hierarchies, and the subsequent health effects from any status differences in women, have been largely ignored to this point in the scientific literature.

## Supplementary material

For supplementary material accompanying this paper visit http://dx.doi.org/10.1017/ehs.2020.44. click here to view supplementary material
