# Supplementary material for: Status does not predict stress: Women in an egalitarian hunter–gatherer society
Source: Evol Hum Sci. 2020 Aug 24;2:e44. doi: 10.1017/ehs.2020.44 (PMC10427491; doi:10.1017/ehs.2020.44)

Supplementary Material

Table 1 Skewness values of Popularity and digging reputation scores

|  | Camp A | Camp B | Camp C | Camp D | Camp E | Camp F | Camp G | Camp H |
| --- | --- | --- | --- | --- | --- | --- | --- | --- |
| Popularity | 0.84 | 0.56 | 0.78 | 0.14 | 1.10 | 1.10 | 0.02 | 0.10 |
| Foraging reputation | 0.85 | 1.57 | 0.001 | 2.50 | 0.51 | 0.79 | 0.17 | 1.29 |

Table 2 LMM results with Popularity excluded.

|  | *B* ± SE | t value | Pr(>\|t\|) | AIC | R^2^ |
| --- | --- | --- | --- | --- | --- |
| Intercept | 3.546 ± 0.50 | 7.06 | <0.001 | 115.4 | Marginal: 0.04 |
| Age (logged) | 0.145 ± 0.13 | 1.36 | 0.177 |  | Conditional: 0.14 |
| Foraging reputation | 0.002 ± 0.06 | 0.03 | 0.976 |  |  |
| Reproductive state | -0.075 ± 0.13 | -0.57 | 0.567 |  |  |

Table 3 LMM results with foraging reputation excluded.

|  | *B* ± SE | t value | Pr(>\|t\|) | AIC | R^2^ |
| --- | --- | --- | --- | --- | --- |
| Intercept | 3.534 ± 0.44 | 8.095 | <0.001 | 115.6 | Marginal: 0.04 |
| Age (logged) | 0.187 ± 0.11 | 1.63 | 0.108 |  | Conditional: 0.14 |
| Popularity | 0.014 ± 0.05 | 0.265 | 0.792 |  |  |
| Reproductive state | -0.072 ± 0.13 | -0.56 | 0.579 |  |  |

In Figures 1a-h, we show the number of individuals in each camp to receive nominations for being the best tuber digger. Higher nomination scores indicate the number of nominations for being the best digger. Number of participants refers to how many people in camp received each nomination score (best digger = 3 points, 2^nd^ best digger=2 points, third best digger = 1 point, not nominated = 0 points).

In Camp 1, for example, four people were never nominated by a single person as being in the top 3 diggers in camp. One person was named so often they had a score of 50.

Figure 1a. Camp 1 Digging nomination breakdown (camp n diggers = 18, n nominators = 33).


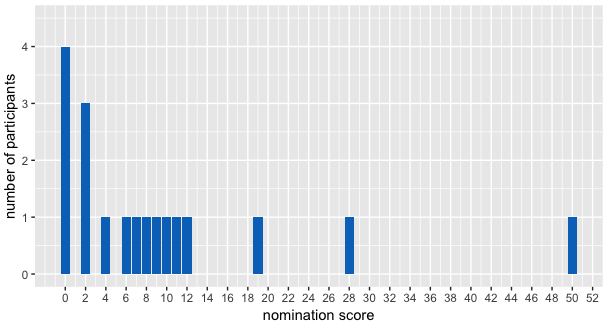


Figure 1b. Camp 2 Digging nomination breakdown (camp n diggers = 19, n nominators =38)


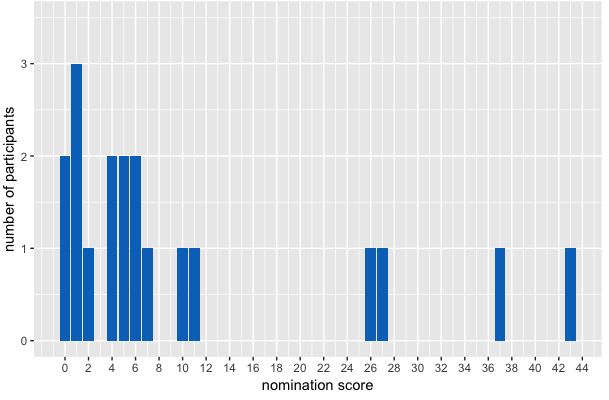


Figure 1c. Camp 3 Digging nomination breakdown (camp n diggers = 7, n nominators = 12)


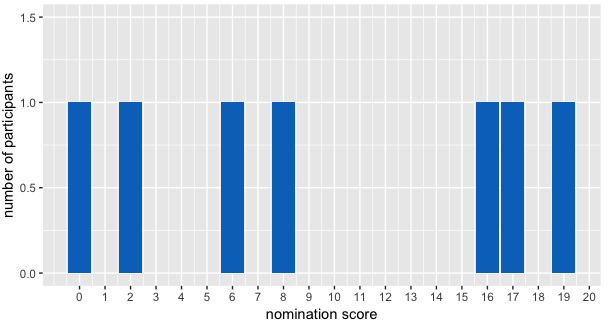


Figure 1d. Camp 4 Digging nomination breakdown (camp n diggers = 14, n nominators =34)


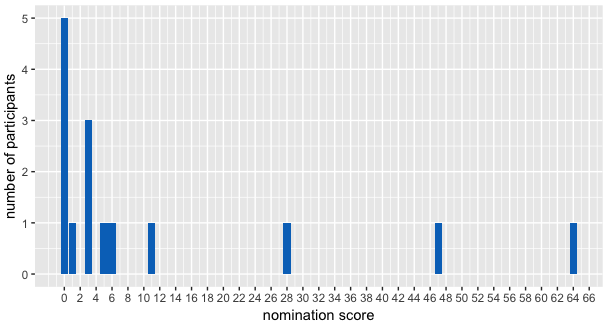


Figure 1e. Camp 5 Digging nomination breakdown (camp n diggers = 3, n nominators = 10)


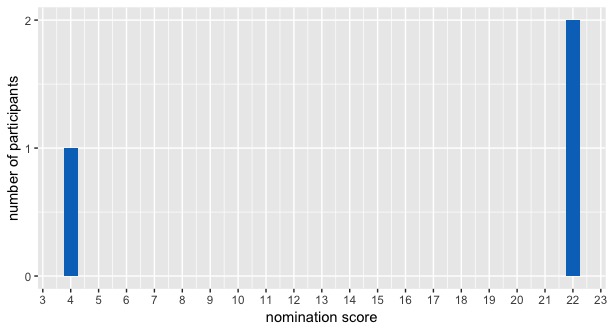


Figure 1f. Camp 6 Digging nomination breakdown (camp n diggers = 4, n nominators = 6)


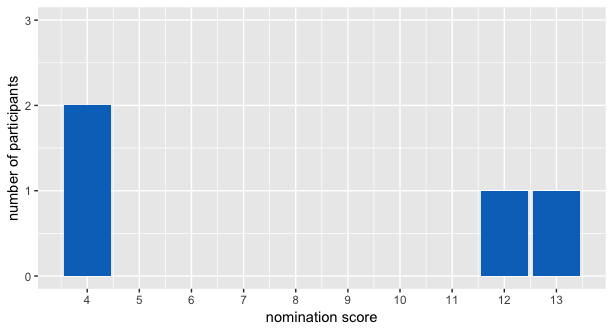


Figure 1g. Camp 7 Digging nomination breakdown (camp n diggers = 3, n nominators = 7)


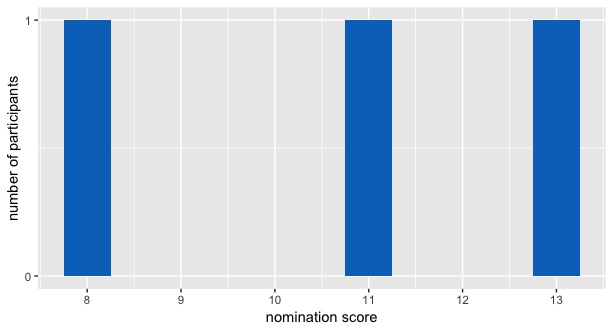


Figure 1h. Camp 8 Digging nomination breakdown (camp n diggers = 10, n nominators = 12)


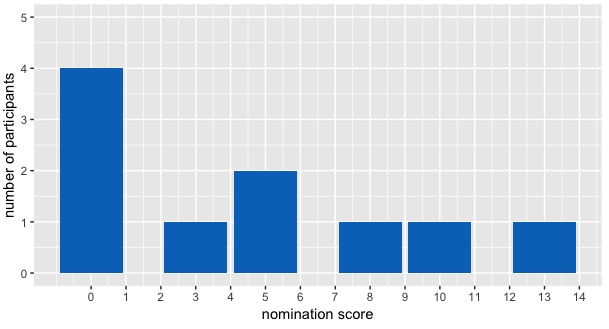


In Figures 2 a-h we show the number of individuals in camp to receive nominations for being the best friends of others. Higher nomination scores indicate the number of nominations for being the among someone’s named three best friends. Number of participants refers to how many people in camp received each nomination score (best friend = 3 points, 2^nd^ best friend =2 points, third best friend = 1 point, not nominated = 0 points).

In Camp 1, for example, four people were never named among anyone’s three best friends, and one person received a score of 15.

Figure 1a. Camp 1 Friendship nomination breakdown (camp n potential friend nominees = 18, n nominators = 33)


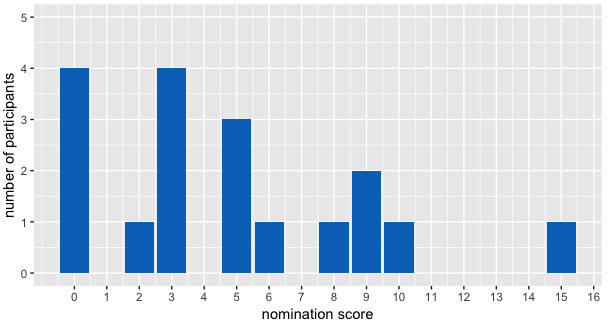


Figure 2b. Camp 2 Friendship nomination breakdown (camp n potential friend nominees = 19, n nominators = 38)


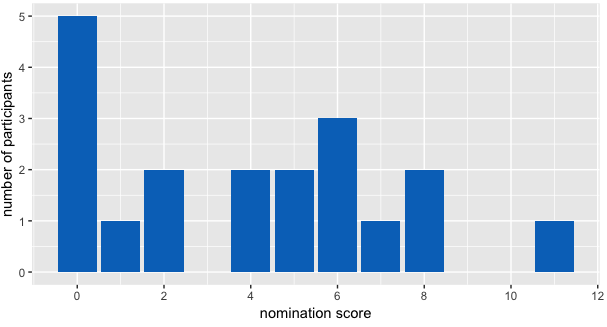


Figure 2c. Camp 3 Friendship nomination breakdown (camp n potential friend nominees = 7, n nominators = 12)


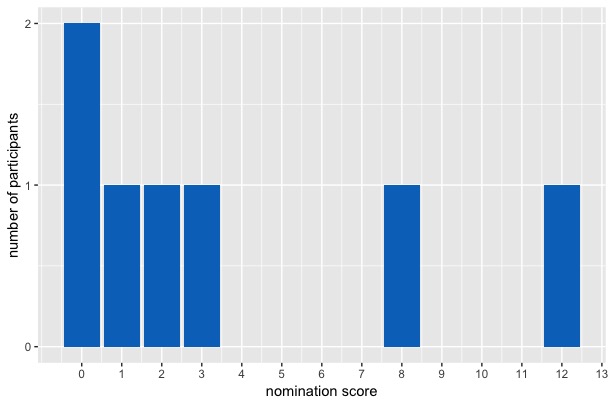


Figure 2d. Camp 4 Friendship nomination breakdown (camp n potential friend nominees = 14, n nominators = 35)


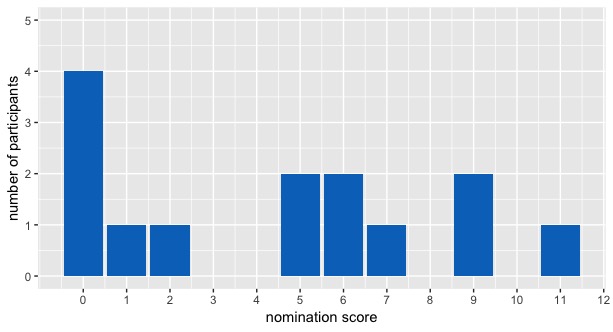


Figure 2e. Camp 5 Friendship nomination breakdown (camp n potential friend nominees =3, n nominators = 10)


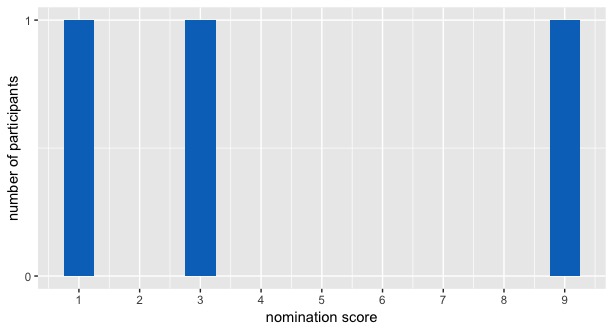


Figure 2f. Camp 6 Friendship nomination breakdown (camp n potential friend nominees = 4, n nominators = 6)


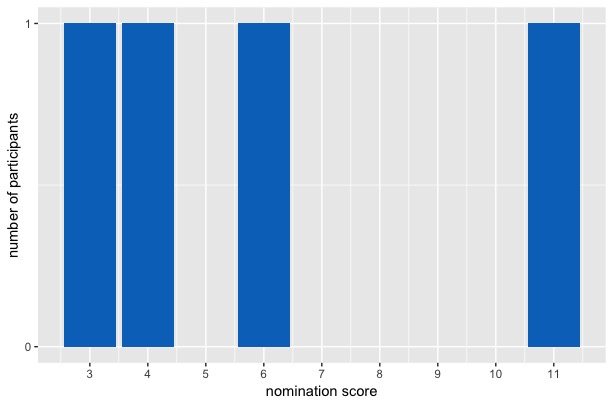


Figure 2g. Camp 7 Friendship nomination breakdown (camp n potential friend nominees = 7, n nominators = 3)


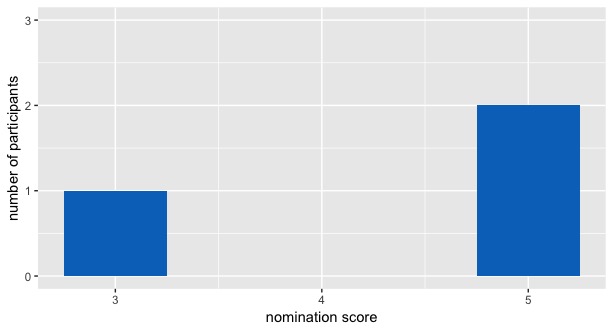


Figure 2h. Camp 8 Friendship nomination breakdown (camp n potential friend nominees = 10, n nominators = 12)


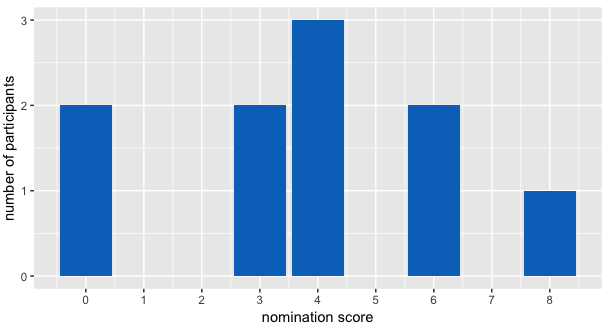

Supplement: Supplementary file 1 [file S2513843X20000444sup001.docx]
